# The Forgotten Tobamovirus Genes Encoding the 54 kDa Protein and the 4–6 kDa Proteins

**DOI:** 10.3390/v16111680

**Published:** 2024-10-28

**Authors:** Peter Palukaitis, Masoud Akbarimotlagh, Sajad Astaraki, Masoud Shams-Bakhsh, Ju-Yeon Yoon

**Affiliations:** 1 Graduate School of Plant Protection and Quarantine, Jeonbuk National University, Jeonju 54896, Republic of Korea; 2 Department of Plant Pathology, Faculty of Agriculture, Tarbiat Modares University, Tehran 14115-111, Iran; masoudakbari001@hotmail.com (M.A.); sajadastaraki1@gmail.com (S.A.); shamsbakhsh@modares.ac.ir (M.S.-B.); 3 Department of Agricultural Convergence Technology, Jeonbuk National University, Jeonju 54896, Republic of Korea

**Keywords:** tobamoviruses, tobacco mosaic virus, tomato mosaic virus, 54 kDa protein, 126 kDa protein, 183 kDa protein, movement protein, P6 protein, ORF6, tobamovirus subgroups, viral protein functions

## Abstract

This article reviews the literature concerning the largely forgotten tobamovirus gene products for which no functions have been ascribed. One of these gene products is the 54 kDa protein, representing the RNA-dependent RNA polymerase segment of the 183 kDa protein translated from the I_1_-subgenomic mRNA, but which has been found only by in vitro translation and not *in plants*. The other is a collection of small proteins, expressed from alternative reading frames (likely from internal ribosome entry sites) in either or both the *movement protein* gene or the *capsid protein* gene. Previously, two small proteins were referred to as the 4–6 kDa proteins, since only single proteins of such size had been characterized from tobacco mosaic virus and tomato mosaic virus genomes. Such putative proteins will be referred to here as P6 proteins, since many new proposed P6 open reading frames could be discerned, from an analysis of 45 of 47 tobamovirus genomes, with a coding capacity of >15 amino acids up to 94 amino acids, whereas other peptides with ≤15 amino acids were not considered here. The distribution of the putative P6 proteins among these tobamoviruses is described, as well as the various classes they fall into, based on their distribution with regard to the organization of other genes in the viral genomes. Models also are presented for possible functions of the 54 kDa protein and the P6 proteins, based on data in the literature.

## 1. Introduction

**Figure 1:**
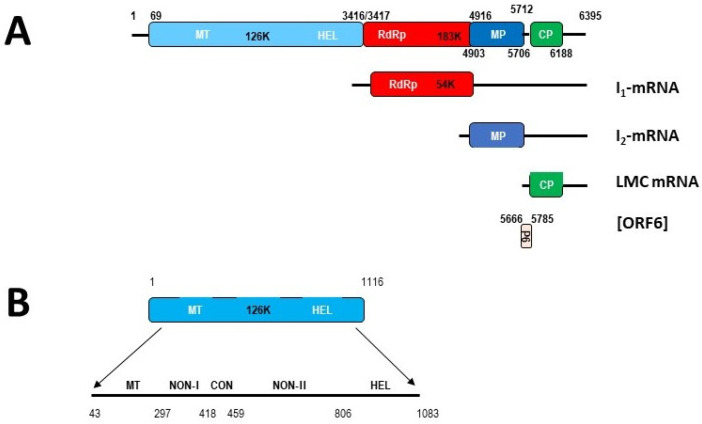
Genome organization and gene expression strategies for tobamoviruses, as determined for tobacco mosaic virus (TMV) and tomato mosaic virus (ToMV). The specific numbers refer to the details for (TMV), the type member of the genus *Tobamovirus*: (**A**) The 126 kDa protein is translated from the viral genomic RNA, as is the 183 kDa protein, the latter by a readthrough of the 126 kDa protein termination signal. The 126 kDa protein contains motifs for the methyl transferase (MT) domain involved in capping of the viral RNAs, and the helicase (HEL) domain involved in binding to numerous protein factors for replication, as well as to the viral RNA. The RNA-dependent RNA polymerase (RdRp) domain is present in the 183 kDa protein, as well as in the 54 kDa protein. The latter has been expressed separately via in vitro translation of the subgenomic I_1_-mRNA. The virus movement protein (MP) is expressed from another subgenomic RNA designated the I_2_-mRNA, while the capsid protein is expressed from a third subgenomic RNA designated the LMC mRNA. Small proteins, designated P6 are proposed to be translated from either the genomic RNA or the I_2_-mRNA, using internal ribosome entry sites (IRES) from one or more open reading frames (ORF) designated ORF 6. The first two proposed ORFs 6 were found to overlap the MP and CP ORFs, but others discussed in this work, are proposed to occur in the MP ORF. (**B**) An expansion of the 126 kDa protein to show the locations of various regions, including the proposed MT domain, the HEL domain, and the interdomain region. The latter is split into three subregions designated Non-conserved I (NON-I), Conserved (CON), and NON-II, with regard to the level of conservation of sequences between tobamoviruses.

The genome map of tobamoviruses, determined first in its family type member, tobacco mosaic virus (TMV) usually depicts four genes (Figure 1A) [1], encompassing the 5′-proximal open reading frame (ORF) encoding both the 122–130 kDa protein (hereafter, 126 kDa) and a second protein of 178–183 kDa (hereafter, 183 kDa). The 183 kDa protein results from readthrough of the amber termination present at the end of the ORF encoding the 126 kDa [2] of all tobamoviruses sequenced to date (these two proteins together comprise the viral-encoded elements of the viral replicase [3,4,5]). Two other ORFs are found at the 5´-ends of two subgenomic (sg) mRNAs produced during virus replication [6,7,8,9,10,11,12,13,14,15]. These mRNAs encode the ca. 30 kDa movement protein (MP) [15,16,17,18,19] and the 17.5 kDa capsid protein (CP) [8,9,10,11,12,13,15] (Figure 1). There are differences among various tobamoviruses that have led to arranging them into subgroups, first on the basis of whether the origin of assembly (OAS) was in either the MP ORF, or the CP ORF [20,21], and subsequently, on whether the ORF for the 183 kDa protein ended before or overlapped with the beginning of the MP ORF, as well as whether the MP ORF ended before or overlapped with the beginning of the CP ORF [22,23,24,25]. These four proteins are translated either directly from the genomic (g) RNA (to produce the 126 kDa protein and the 183 kDa protein), or from sgRNAs designated the I_2_-(Intermediate 2) mRNA, for production of the MP, and the LMC (Low-Molecular-weight Component) mRNA, for production of the CP [2,9,10,11,12,13,14,15].

The 126 kDa protein contains an N-terminal-proximal, methyl transferase (MT) domain (involved in capping of the viral RNA) [26,27] and a C-terminal-proximal, helicase domain (involved in virus replication, oligomerization of the 126 kDa and 183 kDa proteins, and interaction with the tobacco (*Nicotiana tabacum*) *N* resistance gene product) [28,29,30,31,32], as well as three, short, interdomain regions (NON-I, CON, and NON-II; for Non-conserved-I, Conserved, and Non-conserved-II) [33] (Figure 1B). The NON-II and helicase domains, as well as part of the MT domain, individually function as RNA silencing suppressors [34]. The 183 kDa protein contains the viral RNA-dependent RNA polymerase (RdRp) component of the viral replicase, in its C-terminal 56 kDa extension of the 126 kDa protein [3,5,33,35,36,37] (Figure 1A).

These two replication-related proteins expressing from the gRNA, together with the MP and CP, expressed from two sgRNAs, appear to be sufficient genetic material and protein functions for most tobamovirus researchers, such that they have excluded the two other ORFs, which were characterized initially some decades ago. These ORFs encode a 54 kDa protein [38,39] and 4–6 kDa proteins, designated henceforth as P6 proteins [40,41,42,43,44,45,46,47] (Figure 1A). These have been excluded, probably because (i) their functions are not known; (ii) they cannot be detected in planta (54 kDa protein [15,37,39,48,49,50,51,52]), (iii) they are detected with difficulty (P6 proteins [43,45]), or (iv) the ORFs are absent from some tobamoviruses (P6 proteins [45,46]). The small sizes of the P6 proteins and limited sequence similarities among tobamoviruses also militate against their being considered bona fide proteins [44,45,46]. Here, we will review the work on those proteins, and the reasons for believing that they are, in fact, real proteins, and then propose potential roles for these proteins.

## 2. The 54 kDa Protein

In the characterization of the encapsidated RNAs of the tobamovirus sunnhemp mosaic virus (SHMV) [10,11,13], known variously at that time as the legume strain, cowpea strain, or bean strain of TMV, four classes of encapsidated virions were described: the full-length particles (referred to as “L” for Large), the I_1_-particles, the I_2_-particles, and the LMC particles [10,11,13,14,15]. The broader size of the I_1_-class suggested that it might contain broken rods [11,14]. Further work examined these classes of particles for SHMV, the common (U1) strain of TMV, and a wheat strain from Kansas (K-TMV) [14]. That work showed that I_1_-RNA translated to yield two proteins of similar size (29 kDa and 30 kDa); the same as seen for the I_2_-RNA. The authors also showed that the LMC particles did not exist for strains U1 and K, but only for SHMV (at that time) [14]. In addition, the fractionation and translation leading to various other (probably incomplete) translation products (from contaminating L-RNA) with the I_1_-RNA led to focus on the I_2_-RNA, ignoring the I_1_-RNA.

Interestingly, in TMV-infected plant tissues, RNAs radiolabeled in the presence of Actinomycin D (to reduce host RNA synthesis), showed the presence of an RNA of ~1.1 × 10^6^ Daltons (equating to ~3400 nt), similar to the size of the I_1_-RNA, not present in radiolabeled healthy plants [12]. [All estimates from that and earlier times were based on various markers for which the mol. wt. were often themselves determined based on other comparisons, as neither the RNAs nor proteins had been sequenced. Hence, in different publications, the sizes of both proteins and RNAs changed, as these various genes were sequenced].

The second chapter in the I_1_-RNA story involves the characterization of double-stranded (ds) RNAs produced during infection by TMV [53]. These dsRNAs were isolated by cellulose column chromatography, in which various RNAs could be separated from each other based on their elution profiles from CF-11 cellulose: all RNAs would bind in buffer plus 35% ethanol; single-stranded (ss) RNAs could be eluted with buffer plus 15% ethanol; and dsRNAs could be eluted with buffer alone (or just water) [38,53]. Agarose gel electrophoresis of the dsRNAs isolated from TMV-infected plants showed four dsRNAs. All four dsRNAs had sequences corresponding to the 5´-end of the (−) TMV RNA [53]. The largest of these dsRNAs corresponded to the L-RNA, while the second dsRNA corresponded to that of the 1.1 × 10^6^ Daltons RNA described by Siegel et al. [12], although I_1_-RNA was not mentioned. The third dsRNA corresponded roughly to that of the I_2_-RNA, whereas the smallest RNA was considered too small to correspond to the LMC [53]. [This was due to the lower mol. wt. markers not being in a linear range with the larger RNAs.] Since the three smaller dsRNAs were present at much lower levels of accumulation than the largest dsRNA, additional experiments were conducted to establish that it was extremely unlikely that these smaller RNAs were artifacts of isolation [53].

A further characterization of the various ssRNAs and dsRNAs was performed by molecular hybridization analysis using (+) and (−) polarity probes to either the entire TMV genome, or to selected regions of the genome, including the 3′-terminal 1000 nt, the 5′-terminal residues 2–71, and sequences proximal to the OAS (i.e., 47 nt within the MP ORF). This showed that there were four ssRNAs associated with polyribosomes that corresponded in size to the denatured dsRNAs [38]. Moreover, only the largest dsRNA and ssRNA contained the sequences of the 5′-terminal non-translated region (NTR), whereas all RNAs contained the sequences of the CP ORF and 3′-terminal NTR, and only the smallest ssRNA and dsRNA did not have the sequences of the OAS [1,20,21,54,55]. Hence, the I_1_-RNA was again resurrected as a third TMV sgRNA, and it was speculated that this RNA could express the readthrough protein of the 183 kDa protein, as a separate 54 kDa protein [38].

A follow-up study demonstrated that the I_1_-RNA, enriched from TMV RNAs extracted from purified virions, translated to produce an ~50 kDa protein, as well as an ~30 kDa protein (from cross-contaminating I_2_-RNA) [39]. Using RNAs isolated from dissociated polyribosomes, the 5′-end of the I_1_-RNA was mapped to position 3405 in the TMV genome (13–15 nt before the amber termination codon of the 126 kDa ORF), with the nearest downstream AUG at position 3495–3497, in-frame with the 183 kDa protein, and this ORF co-terminating with the 183 kDa ORF at positions 4917–4919, to produce a 54 kDa protein [39] (Figure 1A). Sulzinski et al. [39] also discussed a number of earlier publications in which an ~50 kDa protein was observed lurking in the background of various translations in different systems, often without comment from those authors. On the other hand, numerous other publications using TMV-infected plant or protoplast systems had not demonstrated the presence of an ca. 50 kDa protein, as detailed by Sulzinski et al. [39], nor did a later study using antibodies to the 183 kDa protein [51].

To identify a role for the 54 kDa protein, the Zaitlin lab generated transgenic tobacco plants expressing the 54 kDa ORF [48]. These plants expressed the mRNA for the 54 kDa protein but also did not show the presence of the 54 kDa protein. In addition, these plants were extremely resistant to infection by TMV at high inoculum doses [48], unlike in CP-mediated protection [56,57]. However, the 54 kDa ORF-expressing plants were not resistant to infection by either tomato mosaic virus (ToMV; aka tomato-strain of TMV; aka L-strain of TMV, with ~80% nucleotide sequence identity to TMV [58]) or tobacco mild green mosaic virus (TMGMV; aka U2-strain of TMV, with ~64% nucleotide sequence identity to TMV [59]). These plants did not show either recovery from infection, or infection overcome by using RNA as an inoculum instead of a virus, as often happened with CP-mediated resistance against other viruses [48,56,57]. Subsequent work showed that the replication of TMV in the 54 kDa transgenic plants was not completely inhibited, but was highly suppressed, both in TMV-infected protoplasts made from 54 kDa transgenic plants, as well as in TMV-inoculated leaves; with no infection detectable in upper non-inoculated leaves [49]. Moreover, this same study indicated that the mechanism of resistance was not by inhibition of the (+)-strand RNA synthesis from the (−)-strand RNA, or vice versa, nor did the (in vitro) translation of the 54 kDa protein inhibit the translation of either the 126 kDa or the 183 kDa protein [49]. A further study using transient expression of the ORF encoding the 54 kDa and two different mutants of this sequence, showed that the high level of resistance was associated with the 54 kDa protein and not the encoding RNA [50]. That is, a frameshift mutation producing about 20% of the N-terminus of the 54 kDa protein (yielding a putative protein of less than 14 kDa) did not provide resistance to infection by TMV, whereas, mutation of the initiation codon, which resulted in initiation at the next methionine codon, 14 amino acids downstream, yielding a protein of 50–52 kDa, still provided strong resistance to TMV. In all cases, the accumulation level of the transiently expressed 54 kDa protein-encoding mRNA was comparable, showing that the strong resistance was not RNA-mediated [50].

A different approach by the Culver lab [52], added further information concerning the nature of the resistance. Those authors expressed nine overlapping fragments of sequences encoding parts of the TMV 183 kDa protein (ranging from 713 to 1070 nt, each with its own initiation and termination codons) from a potato virus X (PVX) expression vector in *N. benthamiana*. They found that while three overlapping fragments, completely within the 54 kDa protein-encoding RNA region, gave strong resistance to TMV infection, other constructs expressing sequences of the MT or helicase protein-encoding regions gave only weak resistance to infection by TMV, as expected for RNA silencing. Moreover, frameshift mutants in the three overlapping fragments of the 54 kDa protein-encoding sequences, gave only weak resistance, as also observed for the non-mutated MT and helicase regions. By contrast, frameshift mutations in the six other overlapping fragments, covering the MT and helicase domains, including one fragment also containing the first 207 nt of the 54 kDa protein-encoding sequences, did not show any appreciable difference in resistance from the corresponding non-frameshifted fragments [52]. However, the fragment (number 9) covering sequences encoding the C-terminal half of the 54 kDa protein did not accumulate any protein, unlike all the other fragments, including the ones covering sequences encoding the N-terminal half of the 54 kDa protein (fragment 7) or fragment 8, which overlapped fragments 7 and 9 [52]. These data showed that the intact reading frame of the 54 kDa ORF was required to produce a much higher level of resistance, while the sequences from other regions of the 183 kDa ORF gave rise to only a weak resistance, which was not protein-mediated but rather RNA-mediated, via RNA silencing. In the absence of an intact in-frame ORF, the sequences of the 54 kDa protein-encoding region also could function in RNA silencing [52]. The absence of a detectable 54 kDa protein in plants was not mirrored in yeast cells, where the region corresponding to the 54 kDa protein (corresponding to amino acids 1205–1613 of the 183 kDa protein) was expressed stably for yeast two-hybrid analyses [37].

Various authors have suggested that the 54 kDa protein may have some function in virus replication, involved in the conversion from (−) strand synthesis to (+) strand synthesis, the synthesis of sgRNAs vs. gRNA, or as a repressor of further replication, and that the expressed 54 kDa protein must be unstable [15,48,49,50,60]. The symmetrical replication of both (+) and (−) strands of TMV RNA, both at low levels, in 54 kDa ORF transgenic tobacco plants [50], suggests that the 54 kDa protein is not involved in the switch from (−) to (+) RNA synthesis. We cannot rule out that it may have a role in sgRNA synthesis, but such a role would likely be to inhibit sgRNA synthesis to maximize gRNA synthesis, since purified TMV gRNA produced sgRNA without the I_1_-RNA included in the inoculum [14,38,53]. Rather, we suggest a different role and “fate” for the 54 kDa protein. This proposed role involves the 54 kDa protein functioning as a decoy to protect the viral RdRp from a host protease-mediated degradation system (PDS). Before explaining this model, it is necessary to review the literature on the role of host factors from the PDS and their involvement in viral infection cycles.

A number of host factors have been identified that are involved in TMV replication (reviewed in [61,62]). These include a TMV-associated RING finger protein (TARF) [63]. TARF is a 138 kDa protein, containing 1233 amino acids, with three paired HHE (Hemerythrin) domains (amino acids 46–107 plus 115–176, 304–366 plus 372–435, and 647–708 plus 718–807), a putative CHY-type zinc-finger (ZF) domain (amino acids 988–1965), and a putative RING-finger (RF) domain (amino acids 1102–1165) [63]. TARF was identified via interaction in the yeast two-hybrid system of an *N. tabacum* cv. Xanthi cDNA library was screened using the RdRp region of the 183 kDa ORF as bait [63]. The HHE domain is conserved in an oxygen transport protein [64]; the CHY-type ZF has no known function, but is conserved in Pirh2, a eukaryotic E3-ubiquitin ligase, containing three ZFs adjacent to an RF domain [65], whereas the RF domain is conserved in numerous E3 ubiquitin ligases [66,67,68]. TARF fused at its C-terminus to GFP was distributed in the nucleus, the cytoplasm, and associated with the cytoskeleton, of tobacco cells [63]. The protein expressed in yeast from the original TARF clone, representing only the C-terminal 263 amino acids, bound 6–7 times more strongly to the 54 kDa sequence than did the full-length TARF clone, suggesting some interference from other regions of TARF outside the ZF and RF domains; neither TARF protein interacted significantly with the MT, helicase, or interdomain region [63]. TMV infection induced *TARF* gene expression: the highest level was at the first time point, 4 h post-infection (hpi) after the zero time point, with a rapid decrease to 12 hpi, a low expression level until 48 hpi, and no significant expression at 96 hpi [63]. Silencing expression of the *TARF* gene, using virus-induced gene silencing (VIGS) with the aid of a PVX vector, resulted in more than 5-fold increase in TMV accumulation at 1–2 days post inoculation (dpi), whereas transient overexpression of *TARF* in *N. benthamiana* by agroinfiltration led to an ~8-fold reduction in TMV accumulation [63]. Intriguingly, infection of the *TARF*-silenced leaves, with TMV-GFP, showed more infection foci (~4-fold), with the lesions approximately the same in area, at 7 dpi, whereas transient overexpression of *TARF*, followed by infection with TMV-GFP, led to a similar number of lesions, but with approximately one-third the area vs. plants infiltrated with an empty vector [63]. Yamaji et al. [63] suggested the former (*TARF*-silencing) result indicated that TARF negatively regulated an early stage in TMV replication, rather than virus movement, whereas the latter (*TARF*-overexpression) result indicated that TARF negatively regulated TMV accumulation [63]. Yamaji et al. also suggested that the different effects between silencing and overexpressing TARF might relate to the need for other factors to coordinate the expression of TARF (functioning as an E3 ubiquitin ligase) along with ubiquitin-activating enzyme (E1) and ubiquitin-conjugating enzyme (E2), resulting in a higher level of inhibition [63]. Previously, two forms of tobacco *E1* (*NtE1A* and *NtE1B*) had been shown to be induced by infection with TMV and ToMV [69]. By contrast, either suppression of tobacco *Ubiquitin* gene expression or expression of a mutant *Ubiquitin* gene (which could not support polyubiquitination of monoubiquitinated proteins, necessary for targeting the 26S proteasome [70,71,72]), resulted in the inhibition of TMV infection in tobacco [73]. In addition, silencing the F-box protein ACIF1 (associated with a class of E3 ubiquitin ligases called SCF [70]) led to the suppression of TMV helicase-mediated activation of the *N* gene-mediated resistance response [74]. These results showed that the ubiquitin-mediated, proteasomal degradation system (UPS) is involved in regulating TMV infection.

In a study from the Beachy lab examining the instability of the TMV MP in tobacco protoplasts, the authors also found that the UPS is involved in ubiquitination and degradation of the MP [75], and that the stability of the TMV CP showed no decrease in accumulation over 72 hpi. However, the 183 kDa protein showed different kinetics of accumulation in the presence of either an inhibitor of lysosomal proteases (ALLM), or inhibitors of the 26S proteasome degradation pathway (MG115 or *clasto*-lactacystin-β-lactone) [75]. Specifically, ALLM increased the accumulation of the 183 kDa at the earliest time point (10 hpi), while also showing higher 183 kDa levels at the later time points vs. the control, whereas the two 26S proteasome inhibitors also increased the accumulation at 10 hpi, but then showed similar degradation profiles to the control [75].

Other studies also have suggested the regulation of plant viral replicases by either the UPS or some other PDS (reviewed in [70,71,72]): (1) The yeast Nedd4-type Rsp5p ubiquitin ligase, which interferes with the replication of the tombusvirus tomato bushy stunt virus in yeast, is required for the degradation of the replicase protein p92^pol^, by either or both the endosome/vacuole pathway and the autophagosome pathway, but not the UPS pathway [76]. (2) An *N. benthamiana* E3 ubiquitin ligase containing a RING domain, designated NbUb3R1, was shown to be involved in the inhibition of replication of the potexvirus bamboo mosaic virus, by interaction with the RdRp domain of the viral replicase [77]. (3) Ubiquitinylation of the 66 kDa RdRp of the tymovirus turnip yellow mosaic virus (TYMV) was also described [78]. TYMV gRNA is translated into a 206 kDa protein, which is self-processed to a C-terminal 66 kDa protein containing the polymerase domain and an N-terminal 140 kDa protein; the latter containing an MT at its N-terminus, a papain-like cysteine protease (Pro) domain in the middle, and an NTPase/helicase domain near its C-terminus [79,80]. The 140 kDa protein is rapidly processed to an N-terminal 98 kDa fragment containing the MT and Pro domains and a C-terminal 42 kDa NTPase/helicase domain [80]. Replication requires interaction between the 66 kDa RdRp protein and the membrane-bound 140 kDa protein, to target the RdRp to the chloroplast envelope [79,81,82]. During replication, the polymerase is reduced in accumulation [79], due to degradation by the UPS [83]. This degradation is not complete, since the Pro also inhibits degradation by the UPS [84]. Thus, there seems to be a point in the replication cycle of some viruses at which replication is reduced by degradation of the polymerase.

**Figure 2:**
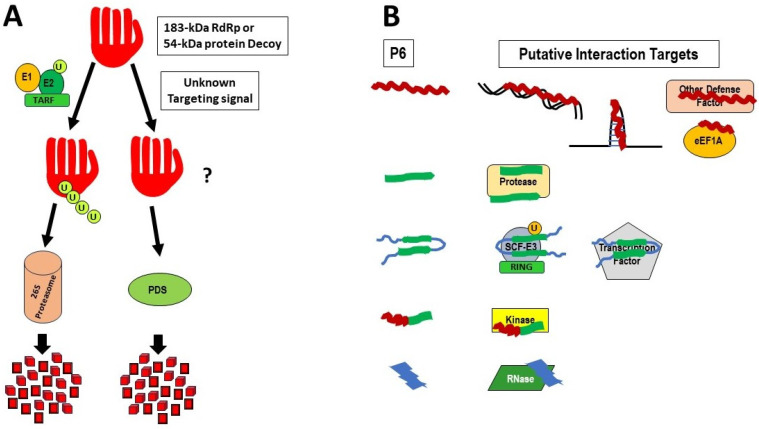
Models for possible roles of the 54 kDa protein (**A**) and the P6 proteins (**B**) during infection: (**A**) The 54 kDa protein is depicted as a right-handed structure, as described for various RdRps, with the thumb, palm, and fingers representing different substructural regions of the RdRp [85]. As described in the text, the 54 kDa protein may serve as a decoy to prevent the RdRp in the 183 kDa from being degraded, either (left side) by the TARF (TMV-associated RING Finger protein) complex and the 26S proteasome [the ubiquitin-mediated, proteasomal degradation system (UPS)], or (right side) by some other protease-mediated degradation systems (PDS), mediated by unknown targeting signals. TARF is an E3 ubiquitin ligase, acting in concert with ubiquitin-activating enzyme (E1) and ubiquitin-conjugating enzyme (E2), to polyubiquitinate proteins as signals for destruction. (**B**) The P6 proteins could act as countermeasures to inhibit specific host defense proteins (or interact with other factors, including DNA and RNA) that may have either regulatory or inhibitory roles in the infection cycle of tobamoviruses. The P6 proteins (left side) are shown as representing the various classes of antimicrobial peptides (AMPs), whose structures are classified as α-helices (α; in red); β-sheets [β; in light green, alone or in more complex arrangements including antiparallel β-sheets connected and flanked by random coils (blue)]; combined α-helices and β-sheets (αβ); or non-αβ (blue) [86]. Possible interacting targets include RNA, DNA, other defense factors, eukaryotic elongation factor 1A (eEF1a), proteases, other E3 ubiquitin ligase complexes, transcription factors, kinases, and specific RNases. In the absence of experimental data, the specific associations of particular structures with specific factors are for modeling purposes only.

We propose that the absence of detectable 54 kDa protein is due to a degradation activity, targeting the 54 kDa protein to a PDS. Given the effects of silencing or overexpressing TARF (an E3 ubiquitin ligase [63]) on the infectivity of TMV in tobacco, the induction of NtE1A and NtE1B (E1 ubiquitin-activating enzymes [69]) by TMV in tobacco, and the effects of various protease inhibitors on the stability of the TMV 183 kDa protein in tobacco protoplasts [75], we propose that the 54 kDa protein is degraded by one or more PDS. We also propose that the 54 kDa protein is not directly involved in replication, but rather acts as a decoy for the PDS, nullifying a host defense against tobamovirus replication by targeting the RdRp component of the viral replicase (Figure 2A). This decoy countermeasure would allow the viral replicase to form and function, largely “unmolested” by the PDS. Transgenic expression of the 54 kDa protein could stimulate the expression of TARF, as well as E1 and E2, prior to infection by the corresponding tobamovirus, leading to a strong, but incomplete, inhibition of virus replication early during infection [49]. This process could still leave a lower, but significant resistance mediated by RNA silencing, as seen in [52]. That RNA silencing, which does not function against either ToMV or TMGMV [48], would allow those viruses to accumulate to normal levels after some slight delay, to overcome the transgenic TMV 54 kDa activation of the PDS. The effect of reduction in TMV replication also would have a carryover effect in reducing the accumulation of 54 kDa protein, from lower levels of accumulating TMV gRNA as well as greatly reduced TMV I_1_-RNA levels. Determining which specific PDS is involved and whether ubiquitinylation, phosphorylation, or both are required, would make an interesting PhD project.

## 3. The P6 Proteins

**Figure 3:**
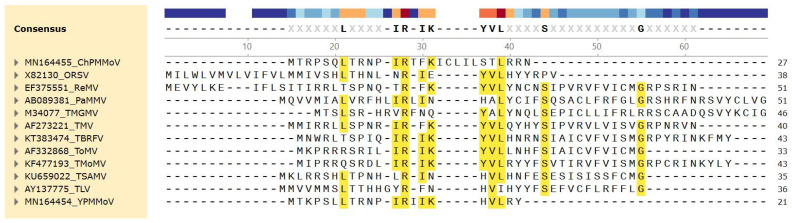
Amino acid sequences and alignment of P6 proteins encoded by Type A (canonical) ORFs 6, present in most Subgroup 1 tobamoviruses and two Subgroup 2 tobamoviruses (PaMMV and TSAMV) (see text). The abbreviations of the 12 tobamoviruses with Type A ORFs 6 are indicated on the left side, together with the GenBank numbers used for the sequence alignments. The conserved sequences are shown highlighted in yellow, and the consensus sequences are shown near the top in bold.

The protein products of what was once called ORF-X [40,41] and later ORF6 [42,43,44,45,46,47], which encode a range of small proteins, varying in size from 4 to 6 kDa, are referred to here as P6 proteins, since there are some additional proposed ones that are much larger than 4–6 kDa and others that are much smaller (see below), while the corresponding ORFs are referred to as ORF6. The first ORF6s described were based on an examination of sequences of various tobamoviruses determined by the early 1990s. These small ORFs encoding putative polypeptides of 39, 33, and 45 amino acids, in U1-TMV, L-ToMV, and TMGMV, respectively, overlapped the region containing the end of the MP ORF and beginning of the CP ORF, but in different reading frames from either the MP or CP ORFs [40]. The genomes of three other tobamoviruses, SHMV, cucumber green mottle mosaic virus (CGMMV), and pepper mild mottle virus (PMMoV) contained ORF6-like sequences, but without the AUG initiator codon. Those authors also suggested that these P6 proteins contained significant sequence similarity [40], but this did not hold up as more ORF6 regions were discovered in other less-related tobamoviruses [(see Figure 3)]. Morozov et al. [40] also showed that an RNA synthesized from a construct containing the L-ToMV ORF6-coding sequences, translated in three different cell-free systems, could produce a 4 kDa protein, as well as a larger 54 kDa protein complex, later shown the be a host ca. 50 kDa protein binding strongly to the P6 protein [41]. [This complex is not to be confused with the 54 kDa protein expressed from the viral genome.] Neither translation product was generated if the plasmid containing a cDNA clone of ORF6 was treated before transcription, with a restriction enzyme (*Hin*fI) that cut inside ORF6 after the sequences encoding the 8th (or 10th) amino acid (in both TMV and L-ToMV), whereas the 54 kDa complex could only be dissociated by treatment of the wildtype (WT) construct translation product with 8 M urea, prior to gel electrophoresis [40] (their unpublished results). Moreover, after transcription in vitro, the sequences downstream from the *Hin*fI sites, when expressed from a plasmid containing the TMV 5′ NTR leader sequence (containing the initiator AUG) were still able to generate the 54 kDa translation product complex in the absence of the original P6 N-terminal 8 or 10 amino acids. This indicated that the largely alternating basic and hydrophobic amino acids in that region (MKPRRRSRIL) were not required for the interaction of the 4 kDa protein with the larger ca. 50 kDa protein [40]. When mutations were made in an overlapping and conserved domain (amino acids 10–14: LIRIK), initially to determine whether the strength of the positive charge was an important factor in the interaction to generate the 54 kDa protein complex, the results demonstrated that the nature of the amino acids (positive or polar, at positions 10 or 12, or negative at position 14) was not important, but the overall change needed to be positive [40]. In addition, the isoleucine at amino acid 11 could not be substituted by threonine [40]. This was verified in a subsequent study, in which the L-ToMV ORF6 with a mutation either solely at isoleucine 11 to threonine or a mutation at the LIRIK quintet to SICIE was examined for the efficiency of forming the 54 kDa protein complex, relative to WT L-ToMV ORF6 [42]. Gushchin et al. found that the single mutation at position 11 reduced the accumulation of the 54 kDa protein complex to about 2–3% of the level given by the WT L-ToMV ORF6, while the triple mutation in the SICIE quintet mutant reduced this accumulation to about 41% [42]. Those authors also extended the analysis to include the ORF6 of a K-strain of TMV. (Not the wheat strain from Kansas [14]); this K-strain differed in sequence from L-ToMV ORF6 by only one amino acid (leucine 18 to proline in K-TMV ORF6, although perhaps this K-TMV was actually a ToMV strain, rather than a TMV strain, given the considerable differences in sequence between the ORFs 6 of U1-TMV and L-ToMV, and the misnaming was a carryover from the days when all tobamoviruses were referred to as strains of TMV.) In this case, mutations converting the five basic amino acids between amino acids 1 and 9 either to an acid amino acid (amino acid 2) or to polar amino acids (amino acids 4–6 and 8) (mutant K-ORF6-RK, containing the sequence MEPTCQSQI) had no effect on the formation of the 54 kDa protein complex. By contrast, mutation of the adjacent LIRIK sequences to SICIE reduced this accumulation to about 37%, and the mutation of K-ORF6 at amino acid 11 from isoleucine to threonine reduced the accumulation to about 20% [42]. The variation between the two sets of results depending on the strain examined may be due to either the large standard error of the data or a structural effect caused by the presence of a proline residue near the middle of the sequence.

Morozov et al. [40] also demonstrated that the 54 kDa protein complex (used to measure the expression of ORF6) could be produced from a TMV-U1 construct in which a truncated MP ORF was present, indicating that ORF6 potentially could be translated from either the genomic RNA or the I_2_-mRNA [40]. In addition, centrifugation of the translation mixture after treatment with different concentrations of KCl showed that 54 kDa protein complexes were associated with ribosomes after treatment with 50 mM KCl (85–90%), or 100 mM KCl (~50%), but poorly so with 500 mM KCl (0–15%) [40]. Subsequently, the ca. 50 kDa host protein interacting with the P6 proteins of both L-ToMV and U1-TMV was shown to be the eukaryotic translation elongation factor 1A (eEF1A) [41], a major cellular protein [87]. Since it was known that wheat germ and *N. benthamiana* eEF1A could bind to two pseudoknot regions in the 3′-NTR of TMV RNA [88], Gushkin et al. [42] examined whether the WT L-ToMV ORF6 translation product and the isoleucine to threonine mutant (L-ToMV ORF6-IT) could bind to tRNA and the U1-TMV RNA 3′-NTR region. They found that both the WT L-ToMV P6 and IT-mutant P6 proteins bound to the two RNAs under a higher protein:RNA ratio, whereas under a lower ratio, the two P6 proteins bound preferentially to the U1-TMV RNA 3′-NTR. Moreover, the WT P6 protein apparently bound cooperatively to both RNAs, forming large complexes, whereas the IT-mutant did not [42]. This indicated to the authors that the cooperative binding in the P6 protein likely involved the same region as was involved in the binding to eEF1A [42]. Hence, Gushkin et al. [42] suggested that the P6 protein may modulate the ability of eEF1A to interact with the pseudoknot region of the 3′-NTR of TMV RNA.

The tobacco eEF1A is also involved in binding to the MT region of the TMV 126/183 kDa proteins in vivo, as well as the TMV 3′-NTR pseudoknot region in vitro [89]. Furthermore, VIGS of the *N. benthamiana eEF1A* genes, using a PVX vector (causing 75–80% reduction in eEF1A mRNA accumulation), resulted in considerable inhibition of TMV accumulation (68–87%) and movement (75–80%) in the infected plant, while not affecting translation of a control gene [90]. This indicates that the e1EF1A protein has a major role in TMV amplification, and thus interference with these functions by the P6 protein could be viewed as a self-regulatory role inhibiting further virus replication. This would then have effects on virus movement, since the viral replication complex is known to be involved in cell-to-cell movement [91].

Although it is tempting to speculate that failure of this inhibitory process could potentially result in excessive replication detrimental to infected cells and the plant as a whole, the evidence from the study of infection by U1-TMV, in the presence or absence [TMV(ORF6-)] of ORF6 expression, did not support this hypothesis. In tobacco (cv. Samsun nn), infection by WT TMV or TMV(ORF6-) did not result in any differences in either pathogenicity (in plants) or in the levels of accumulation of the gRNA and LMC sgRNA (in plants and protoplasts) [43]. While the results were similar in tomato (*Solanum lycopersicum*), there was a reduced level of LMC sgRNA accumulation in TMV(ORF6-)-infected plants [43]. In *N. clevelandii*, infection by TMV(ORF6-) resulted in slightly milder symptoms, as well as a reduced level of gRNA accumulation vs. infection by WT TMV (the LMC RNA was not visible in these plants infected with either WT TMV or TMV(ORF6-) [43]). In *N. benthamiana*, there also was no difference in the accumulation of either RNA or CP after infection by WT TMV vs. TMV(ORF6-); however, there was a major effect on pathogenicity: WT TMV induced a lethal systemic necrosis in infected *N. benthamiana* plants, whereas TMV(ORF6-) caused severe stunting with rugosity and leaf curling in infected *N. benthamiana* plants [43]. Thus, there was no general effect on the replication of TMV by expression or non-expression of the ORF6.

It is conceivable that the lethal pathogenicity effect on *N. benthamiana* by infection with WT TMV is due to the effect(s) of the P6 protein in a host that is considerably debilitated in the expression of a number of defense genes [92]. Overexpression of TMV ORF6 from either a PVX or tobacco rattle virus (TRV) vector in *N. benthamiana* also did not affect the accumulation of the viral gRNAs, but did enhance the pathogenicity induced by the vector viruses [43]. However, in *N. benthamiana* infected by PVX expressing ORF6, the levels of the two PVX sgRNAs increased, but not when infected by WT PVX [43]. In tobacco, PVX expressing ORF6 did not show any effects on either the pathogenicity of PVX or viral RNA accumulation. While TRV expressing either ORF6 or a non-translatable form of ORF6 did not show any differences in accumulation of the viral RNAs, TRV expressing ORF6 induced large necrotic lesions on the inoculated leaves, midribs, and petiole leading to the collapse of the leaves by 4 dpi [43]. This difference in behavior of TMV and PVX vs. TRV in tobacco, vis-à-vis effects of expression of ORF6, was suggested to be due to differences in the RNA silencing suppression activities of TRV [43], since TRV-based vectors are able to induce VIGS more uniformly in *N. benthamiana* than either PVX- or TMV-based vectors [93], although VIGS is not as efficient in tobacco as in *N. benthamiana* [94]. TMV P6 also did not function as either an RNA silencing suppressor or in synergism between PVX and TMV in tobacco [43]. Thus, it appears that the role(s) of P6 may be indirect.

Infection of *N. benthamiana* by either L-ToMV or an L-ToMV mutant with a non-translatable ORF6 [ToMV(ORF6-)] both resulted in severe systemic necrosis leading to the death of the plant [44]. In addition, infection of several other plant species by either L-ToMV or L-ToMV(ORF6-) led to similar symptoms and timing of symptoms by the two viruses, i.e., leaf malformation in *N. clevelandii*, mosaic symptoms in tobacco (cv. Samsun nn), and mosaic plus leaf malformation in tomato [44]. Similarly, a time course of virus accumulation for up to 21 dpi in *N. clevelandii* or tomato showed no difference in viral-specific RNA accumulation; in *N. benthamiana*, the same was true in samplings made until plant death [44]. To examine a differential effect on cell-to-cell movement, Gushchin et al. [44] tested the effects of inoculation of the two L-ToMV variants on hypersensitive tobacco cultivars (Samsun NN and Xanthi NN), which develop necrotic local lesions, around 36–40 hpi. They found no differences in the lesion size developed by the two L-ToMV variants, similar to the results obtained by Canto et al. [43] for timing and size of lesion development in Samsun NN tobacco after infection with U1-TMV and TMV(ORF6-). Hence, ToMV P6 had no detectable effects on viral RNA accumulation, cell-to-cell spread, or pathogenicity. Although infection of *N. benthamiana* by TRV expressing either the L-ToMV ORF6 [TRV(L-ORF6)] or the U1-TMV ORF6 [TRV(U1-ORF6)] resulted in the appearance of leaf curling symptoms and necrotic regions on systemically infected leaves; however, infection by TRV(U1-ORF6) also caused severe stem necrosis leading to death of the stem apex [44], similar to that described in another lab [43] for the expression of U1-TMV ORF6 in a TRV vector. This difference in the pathogenicity of the two ORFs 6 when expressed from a TRV vector, was examined further to map the sequences causing the different pathogenic effects. The eight C-terminal, hydrophobic sequences of L-ORF6 (CVFVICMG) were replaced by the (longer) 14 C-terminal, less hydrophobic sequences of U1-ORF6 (RVLVISVGRPNRVN) in the construct L::U1-ORF6, with the reciprocal exchange involving the eight C-terminal sequences of L-ORF6 replacing the 14 C-terminal sequences of U1-ORF6, in the construct U1::L-OF6. Both hybrid ORFs were then expressed from a TRV vector in inoculated *N. benthamiana* plants, showing that TRV(U1::L-ORF6) induced necrosis on the leaves as with TRV(L-ORF6), whereas TRV(L::U1-ORF6) induced more severe symptoms leading to apical death, as with TRV(U1-ORF6), suggesting that the observed pathogenicity was associated with the C-terminal sequences of the two P6 proteins [44]. However, mutation of sequences encoding seven C-terminal L-ORF6 amino acids (CVFVICM) to sequences more hydrophilic (SASATRT), and expression from a TRV vector, resulted in a mild phenotype similar to that of TRV expressing the green fluorescent protein (GFP) alone. Hence the severe pathogenicity of U1-ORF6 requires additional changes besides the differences in hydrophobicity [44].

A comparison of the subcellular localization of the TMV P6 protein and the ToMV P6 protein, each fused to the yellow fluorescent protein (YFP), along with GFP fused to various subcellular maker proteins and YFP alone (which distributed in the cytoplasm and the nucleus, but not in the nucleolus), each expressed after agroinfiltration into leaves of *N. benthamiana*, showed that at 1–2 dpi, the U1-P6-YFP was found predominantly in the nucleus and concentrated in the nucleolus. At 3–4 dpi, the yellow fluorescence was found mostly in the cytoplasm, in small (0.8–1 μm) aggregates, and further analysis using a mitochondrial marker showed that at 3 dpi, the U1-P6-YFP co-localized in the mitochondria [44]. By contrast, L-P6-YFP was found only with the endoplasmic reticulum (ER) and the nuclear envelope, which did not change over time. When the L::U1-ORF6 and U1::L-ORF6 chimeric constructs, each fused to the *YFP* gene, were infiltrated into *N. benthamiana*, the resulting fluorescence indicated that L::U1-P6-YFP was found mostly in the nucleolus but also diffusely in the nucleoplasm and cytoplasm, and not in the mitochondria, whereas U1::L-P6-YFP was found associated with the ER, but not the nucleoplasm or nucleolus; these associations did not change with time [44]. Hence, the C-terminal regions of the P6 proteins largely affected the distribution of the P6 proteins. Since neither chimeric protein localized to the mitochondria, the latter localization may have required either sequences distributed over different parts of U1-P6, or the association of the L-P6 C-terminal sequences in U1::L-P6-YFP with the ER precluded later movement to the mitochondria [44]. The nucleolar localization signals in both L-P6 and U1-P6 proteins were identified as being in the basic N-terminal 10 amino acids, and mutation of this region did not affect the association of U1-P6-GFP with the mitochondria [44]. Further analysis of the detection of U1-TMV and another isolate (A15) of TMV in infected *N. benthamiana* and *N. clevelandii*, respectively, by immune-specific electron microscopy of infected tissue sections, verified the presence of TMV-P6, predominantly in the nucleus, with much lower levels present in the chloroplast, mitochondria, and cytoplasm [45]. This study also verified the detection in planta of TMV-P6 derived by expression during TMV infection.

An earlier study indicated that ORF6 could be translated in vitro from either the TMV gRNA or a shortened I_2_-like RNA [40]. However, this still left open the question of how ORF6 was translated if there is no specific sgRNA for this protein, when the other known tobamovirus proteins are translated either from the gRNA (126 kDa and 198 kDa) or sgRNAs [54 kDa (I_1_-RNA), 30 kDa (I_2_-RNA), and 17 kDa (LMC RNA)] [15]. A likely mechanism is that ORF6 is translated via an internal ribosome entry site (IRES) inside or upstream of the MP ORF, as shown by Dorokhov and colleagues for the expression of internal proteins [95,96,97,98]. The IRES, located upstream of the putative initiation site, forms one or more stem-loop structures as well as containing a polypurine tract [95,96,97,98,99], which allows ribosomes to bind and initiate translation. However, the efficiency of translation from an inserted IRES was only about 2–4% of that from a capped subgenomic RNA [98]. Nevertheless, since the MP is expressed transiently in the TMV infection cycle and the CP is expressed late during infection, expression from an IRES may offer advantages for an overlapping P6 protein required either early in the infection process, or throughout the process [98].

Studies from Morozov and colleagues [42,45] indicated that canonical ORFs 6 also existed in other tobamoviruses, but were restricted largely to those in Subgroup 1, most of which were first found in solanaceous plants [100]. They also found other, larger, non-canonical ORFs occurring within the MP ORFs of tobamoviruses of other subgroups, including one encoding a protein of 67 amino acids [45], with 5 nt between the terminator of this ORF and the terminator of the MP ORF, in cucumber fruit mottle mosaic virus (CFMMV), and a protein of 94 amino acids, in both CGMMV and cucumber mottle virus (CMoV) [47], starting 145 nt after the initiation codon of the MP ORF and ending 353 nt before the termination codon of the MP ORF.

Subsequently, while studying differences in symptoms caused between two strains of Youcai mosaic virus (YoMV) in *N. benthamiana*, Ju et al. [47] observed that 13 strains of YoMV, as well as two strains of the related viruses, turnip vein clearing virus (TVCV), contained the same ORFs inside the MP ORF, but in a different frame, encoding 39 amino acid putative proteins. In this case, the putative proteins were highly conserved in sequence among strains, although most had at least four amino acid changes. These all were in the same reading frame as the adjacent CP ORF. A 14th strain of YoMV, strain Rg, did not have a termination codon, and hence, this protein would be fused to the CP, as a N-terminal 63 amino acid extension [47]. Those authors also observed that strains of other related viruses, viz., ribgrass mosaic virus (RMV) and wasabi mottle virus (WMoV), had similar conserved putative ORF6-coding sequences, although for shorter P6 proteins (28 amino acids for three strains of WoMV and 23 amino acids for three strains of RMV), also in the CP ORF reading frame [47]. However, our analysis of the same sequences did not detect the methionine initiation codons, but rather TTG in the GenBank sequences of these ORFs in all six viral strains. In the case of WMoV, a TAG termination codon preceded the TTG codon, whereas, in the case of RMV, a TGG codon (for tryptophan) preceded the TTG codon, just as was found upstream of the (TTG codon for) leucine present in the 39 amino acid P6 proteins of YoMV strains. These errors may have been due to some problem with the ORF-search program used by the authors.

Further analysis of the complete genome sequences of 46 unique tobamoviruses (both those accepted as species and those still pending), and the partial sequence of the MP/CP-3′-NTR of tobacco latent virus (TLV), was conducted by the authors of this work, searching for additional ORFs 6 in other reading frames across the MP and CP ORFs (for canonical P6 proteins), inside the MP ORF alone, and inside the MP ORF and the CP ORF, when the former overlapped with the latter (both for non-canonical ORFs 6). We identified putative P6 proteins of various lengths in each virus (Table 1). These ORFs were divided into the above categories, as well as whether they encoded (poly)peptides of either greater than 15 amino acids or less than and equal to 15 amino acids; in the latter case, they usually were not listed in the table. Only two viruses, brugmansia latent virus and frangipani mosaic virus (FrMV), did not contain ORFs that could encode proteins of more than 15 amino acids, although many others that did encode > 15-amino acid putative P6 proteins also contained smaller ORFs. There were 12 viruses that contained canonical ORFs 6 that could encode putative P6 proteins; viz., chili pepper mild mottle virus (ChPMMoV; of 27 amino acids), odontoglossum ringspot virus (ORSV; 38 amino acids); paprika mild mottle virus (PaMMV; 51 amino acids), rehmannia mosaic virus (ReMV; 51 amino acids), TLV (36 amino acids), TMGMV (46 amino acids), TMV (40 amino acids), tomato brown fruit rugose virus (TBRFV; 43 amino acids), ToMV (33 amino acids), tomato mottle mosaic virus (TMoMV; 43 amino acids), tropical soda apple mosaic virus (TSAMV; 35 amino acids), and yellow pepper mild mottle virus (YPMMoV; 21 amino acids) (Table 1). Except for TMGMV, all of these viruses also contained other non-canonical ORFs, all found within the MP ORF; viz., ChPMMoV (of 17, 20, 16, and 46 amino acids), ORSV (22 and 42 amino acids), PaMMV (20 amino acids), ReMV (26 and 25 amino acids), TLV (22 and 18 amino acids), TMV (51 amino acids), TBRFV (22 amino acids), ToMV (19 and 42 amino acids), TMoMV (26, 39 and 62 amino acids), TSAMV (21, 57 and 42 amino acids), and YPMMoV (17 and 28 amino acids) (Table 1). Twenty-one viruses contained only non-canonical ORFs found within the MP ORF (and not fused to another protein); viz., bell pepper mottle virus (of 25, 16, 18, and 36 amino acids), brugmansia mild mottle virus (44, 29, and 24 amino acids), cactus mild mottle virus (CMMoV; 48 and 19 amino acids), clitoria yellow mottle virus (ClYMV; 16 amino acids), CFMMV (47, 24, and 67 amino acids), CGMMV (94 amino acids), CMoV (94 and 18 amino acids), hibiscus latent Singapore virus (HLSV; 20 amino acids), obuda pepper virus (ObPV; 19 amino acids), pepper mild mottle virus (PMMoV; 21 and 27 amino acids), plumeria mosaic virus (PluMV; 24 amino acids), rattail cactus necrosis-associated virus (RCNaV; 19 and 32 amino acids), RMV (19 and 64 amino acids), scopolia mild mottle virus (SMMoV; 35 amino acids), SHMV (27 amino acids), TVCV (39 amino acids), ullucus mild mottle virus (UMMoV; 16 amino acids), WMoV (31 or 35 amino acids), watermelon green mottle mosaic virus (WGMMV; 94 amino acids), yellow tailflower mild mottle virus (YTMMoV; 19 and 24 amino acids), and YoMV (16 and 39 amino acids, excluding the Rg strain mentioned previously) (Table 1). There were 10 tobamoviruses containing non-canonical ORFs 6 inside the MP ORF, as well as another ORF6 inside the MP ORF but also in-frame with and fused to the N-terminus of the CP ORF; viz., hibiscus latent Fort Pierce virus (HLFPV; of 67, 16, and 26 amino acid, not fused to the CP ORF, plus 22 amino acids fused to the CP ORF), hoya chlorotic spot virus (HoCSV; 26, 22, and 31 amino acids, plus 21 amino acids, respectively for the two types of ORFs), hoya necrotic spot virus (HoNSV; 34 and 88 amino acids, plus 20 amino acids, respectively), kyuri green mottle mosaic virus (KGMMV; 31 amino acids plus 21 amino acids, respectively), maracuja mosaic virus (MarMV; 18, 28, and 24 amino acids, plus 14 amino acids, respectively), opuntia virus 2 (OV2; 31 and 18 amino acids, plus 38 amino acids, respectively), passion fruit mosaic virus (PFMV; 24, 26, 33, and 24 amino acids, plus 14 amino acids, respectively), streptocarpus flower break virus (SFBV; 26 amino acids, plus 55 amino acids, respectively), trichosanthes mottle mosaic virus (TrMoMV; 20 and 17 amino acids, plus 12 amino acids, respectively) and zucchini green mottle mosaic virus (ZGMMV; 17 amino acids, plus 75 amino acids, respectively) (Table 1). Finally, there are two cactus tobamoviruses (CTV1 and CTV2) that have not yet been recognized as species but were isolated from a 34-year-old sample considered to be Sammons opuntia virus (aka, opuntia chlorotic ringspot virus, aka opuntia virus), which was thought to be lost. However, as the sequences of CTV1 and CTV2 are only about 67% identical at the nucleotide level and the CP amino acid sequences are only 54% identical and 66% similar, then they are different viruses and hence it is not known which, if either, is the original opuntia chlorotic ringspot virus. Nevertheless, both of these viruses have an MP ORF that extends, in a different reading frame, deep into the CP ORFs, although to different extents. Both viruses also have ORFs 6 that are inside the MP ORF outside the CP ORF, as well as one ORF each inside the MP ORF overlap of the CP ORF; viz., CTV1 (1 protein of 18 amino acids inside the MP ORF, and 1 protein of 20 amino acids inside the MP and CP ORFs), and CTV2 (2 proteins of 29 and 30 amino acids, plus 1 protein of 19 amino acids, respectively) (Table 1).

Which of these ORFs and putative P6 proteins are relevant? This is not known. Is there a size cutoff limit for the functionality of such proteins? Plant peptide hormones can vary from 6 to 66 amino acids in size [101]. Some plant peptides function as antimicrobial peptides, and these vary in size from 11 amino acids for CAPE1 (a CAP-derived peptide 1, from pathogenesis-related protein 1), also with anti-herbivore abilities [102]; 12 amino acids for PIP1 (PAMP Induced secreted Peptide 1) involved in pathogen resistance against bacteria and fungi [103]; 23 amino acids for an endogenous elicitor peptide (PEP) involved in defense signaling [104]; 45–55 amino acids for Defensins, inhibiting fungi and herbivores [105]; to the 60–66-amino acid snakin-2, inhibiting a wide range of microbes [106,107]. It all depends on what structure a short oligo- or polypeptide can form that will facilitate its interactions with other molecules in the cell [101,108]. This would also be true for viral-encoded, short polyproteins. The latter would need to give the virus an advantage in either supporting its basic functions, or preventing the host from subordinating those functions, either directly or indirectly, by use of various defensive measures. These would be selected, within the restraints of maintaining the existing functions of the proteins encoded by the overlapping genes. Obviously, the putative proteins would have to be expressed. This could be by ribosome binding to an IRES, as mentioned previously [95,96,97,98,99], ribosome binding to an AUG in a different optimal structural context, or translational slippage from the MP ORF. Most of these are likely to be less efficient than the translation of the MP and CP ORFs, but given the large number of template molecules (i.e., viral RNA) vs. the number of host antagonistic protein molecules synthesized from much fewer gene copies, the efficiency of production of these peptides may not be an issue. For the same reasons, optimal Kozak translational context for the AUGs also may not be important. In fact, overexpression of such P6 proteins, such as those achieved from either PVX or TRV protein expression vectors, could be detrimental to the host, as observed in two studies [43,44]. And while the lack of conservation of putative P6 protein sequences among very different tobamoviruses may seem a reason for doubt, the range of different P6 protein sequences available and potentially capable of interacting with numerous host targets would be an evolutionary strength. Thus, we propose that P6 proteins also act as countermeasures for inhibiting host proteins (or interacting with other factors) that may have regulatory or inhibitory roles in the infection cycle of tobamoviruses (Figure 2B). Since some of these small proteins are also highly basic (see below), those functions also may include interactions with host DNA or RNA molecules (Figure 2B). To verify such roles would require examining the effects of inhibiting the expression of various putative P6 ORFs in multiple tobamoviruses on the host transcriptome during infection. That information also would offer possible host protein targets for detecting interactions with the putative P6 protein. 

## 4. Relationships Between Subgrouping Tobamoviruses and the Conservation of P6 ORFs

To understand the relationships between different clusters of tobamoviruses and the conservation of the P6 ORFs, one first has to understand the development of tobamovirus phylogeny and the clustering of different tobamoviruses based on different criteria. We will review this literature first to allow the reader to follow the dialectic that follows. 

The initial subdivision of tobamoviruses was made in the early 1980s, based on sequence data limited to less than a handful of tobamoviruses and the observation that the OAS for TMV was in the MP ORF (designated Subgroup 1), whereas the OAS for SHMV and CGMMV was in the CP ORF (designated Subgroup 2) [20,21]. However, subsequently, only a limited number of new tobamoviruses were evaluated for the position of the OAS, using gel electrophoresis of encapsidated viral RNAs, and/or electron microscopy to observe shorter virions; viz., in KGMMV [109], HLSV [110], ZGMMV [111], CMMoV [112], and MarMV [113]. By the mid-1990s, 12 tobamoviruses had been sequenced completely and 11 others sequenced partially, which allowed Lartey et al. [100] to propose that there were three clusters corresponding to those tobamoviruses first discovered infecting solanaceous species (Subgroup 1), those infecting leguminous and cucurbitaceous species (Subgroup 2) and those infecting brassicaceous species (Subgroup 3), with ORSV (infecting orchidaceous species) considered a recombinant virus [100]. Two hibiscus tobamoviruses (HLTV and HLFPV) infecting malvaceous plants [114,115] added another host family to the list, but those authors did not use subgroup numerical characterizations in that study. By contrast, a different study on these two viruses retained their earlier subgrouping [110] in Subgroup II, along with SHMV and the cucurbit-infecting tobamoviruses CGMMV and KGMMV [113], but they did not consider host families of discovery in their analysis. This may have been because Lartey et al. [100] stated that “the family of the host plant does not play a major role in driving tobamoviral sequence evolution.” In the following years, more host family groups were added to the list of family hosts of first discovery; viz., CMMoV, in the family Cactaceae [112]; MarMV, in the family Passifloraceae [113]; FrMV, in the family Apocynaceae [116]; RMV, in the family Plantaginaceae [117]; ReMV, in the family Orobanchaceae [118]; SFBV, in the family Gesneriaceae [119]; and UMMoV, in the family Basellaceae [120]. In addition, more tobamoviruses were described in previous families that once contained only one tobamovirus member; viz., RCNaV [121], OV2 [122], CTV1, and CTV2 in the family Cactaceae; ClYMV (along with SHMV), in the family Fabaceae (the replacement name for Leguminosae); PFMV [123], in the family Passifloraceae; and HoCSV [124], HoNSV [125] plus PluMV [126], all in the family Apocynaceae. In addition, other new viruses were added to the earliest identified host families. During the course of these developments, the numerical subgroup ranking of tobamoviruses was either replaced by a numerical ranking of the host family (or order) groups [117,119,127,128] or lost altogether [112,113,114,115,116,118,120,121,122,123,124,126,129]. What remained was associations based on the phylogeny of the nucleotide sequences, which largely fitted into the host origin family groups. 

In considering the relationships between tobamoviruses, in the first host-plant family of discovery and the nature of the genome organization between the RdRp ORF, the MP ORF, and the CP ORF, i.e., overlap (OL) or non-overlap (NOL), only three permutations were considered before the system broke down: OL (RdRp/MP) and NOL (MP/CP)—the old Subgroup 1, later 1a; NOL and NOL—Subgroup 1b; and OL and OL—Subgroup 2 (based on the OAS in the CP ORF). Even when this was changed to three subgroups, the obvious fourth combination (NOL and OL) was never considered. Perhaps the participants ignored new relationships occurring between the RdRp and MP ORFs because they were dazzled by the differences in the OL between MP and CP, going from NOL, to a short OL, and in some cases to a huge OL; the last being observed in the cactus viruses [46]: CMMoV MP ORF overlapped with the CP ORF by 146 nt (including termination codons), whereas in RCNaV, the MP ORF overlapped the CP ORF by 216 nt; the CTV1 MP and CP ORFs also overlapped by 146 nt, but the overlap in CTV2 was 269 nt. Oddly, OV2 did not have any overlap between the MP and CP ORFs. Hence, this extended MP ORF is not universal to all cactus tobamoviruses. Dorokhov et al. [46] discussed the significance of such overlaps in terms of increasing within-host fitness.

**Figure 4:**
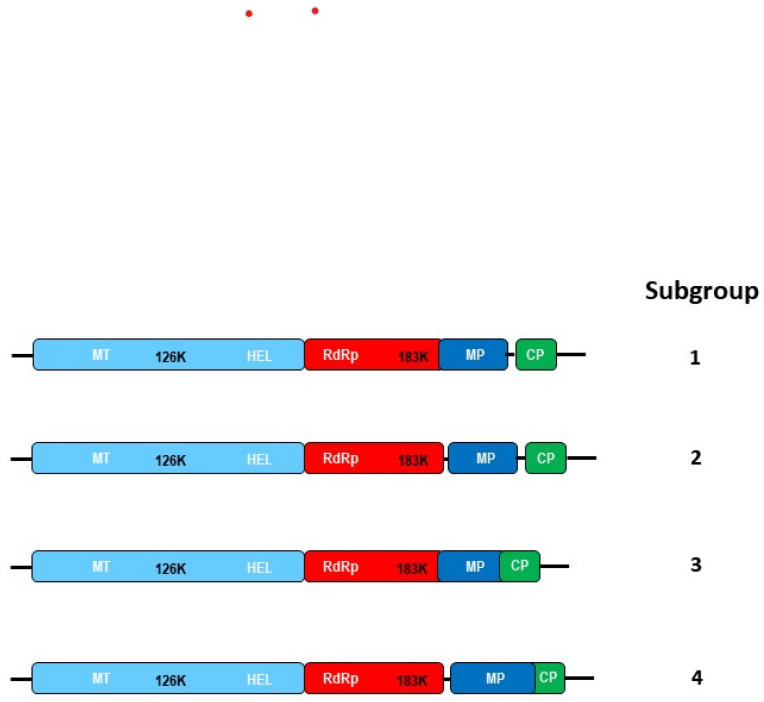
Tobamovirus subgroup designation, based on the MP ORF overlap with either the 183 kDa ORF, the CP ORF, both OTFs, or neither ORF. Subgroup 1 tobamoviruses contain an overlap between the 183 kDa ORF and the MP ORF, but not between the MP ORF and the CP ORF. Subgroup 2 tobamoviruses contain no overlap between any of these ORFs. Subgroup 3 tobamoviruses contain overlaps between both sets of ORFs (183 kDa ORF and MP ORF, as well as MP ORF and CP ORF). Subgroup 4 tobamoviruses contain no overlap between the 183 kDa ORF and the MP ORF, but contain an overlap between the MP ORF and CP ORF, in some cases extended to deep within the CP ORF.

**Figure 5:**
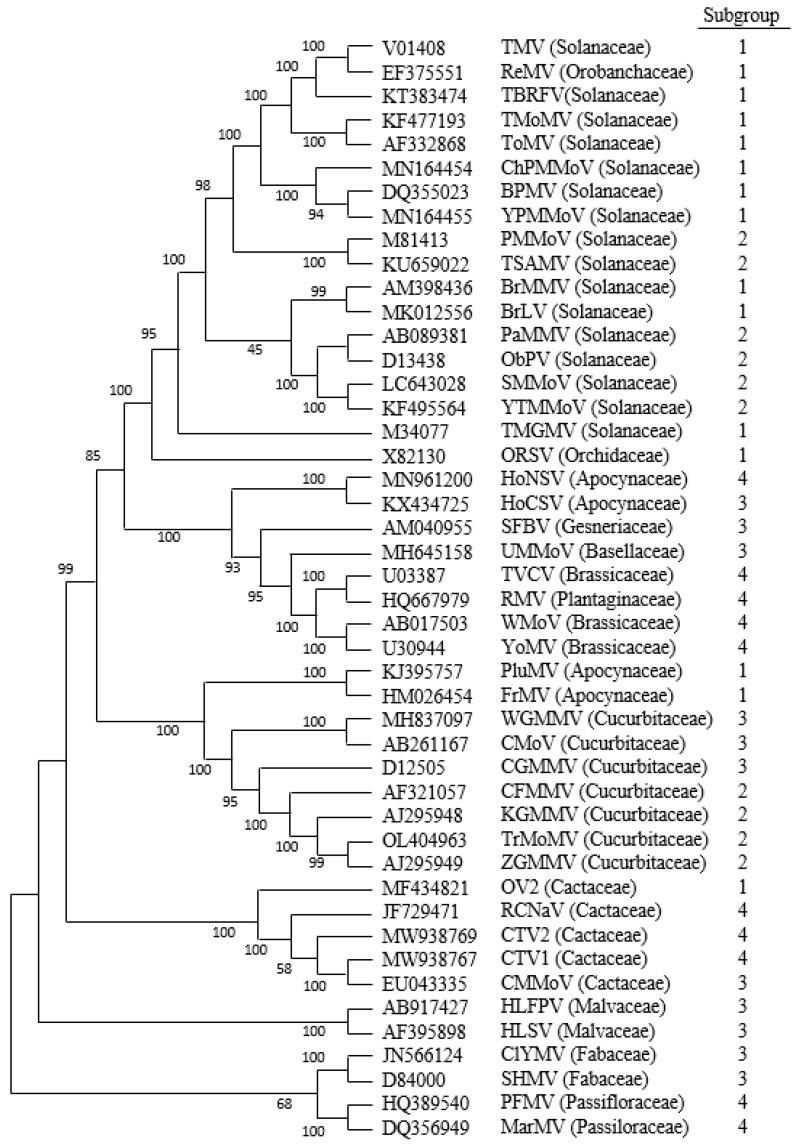
The evolutionary history of tobamoviruses, based on the full-length nucleotide sequence of 46 tobamovirus genomes, was inferred using the Neighbor-Joining method [130], is shown along with the GenBank number and Subgroup number designation of the tobamoviruses, as well as the plant family of first identification of the particular viruses indicated. The percentage of replicate trees in which the associated taxa clustered together in the bootstrap test (1000 replicates) are shown next to the branches.

Therefore, we re-examined the division between tobamoviruses based on four types of OL/NOL situations: those viruses containing RdRp/MP OL and MP/CP NOL are in Subgroup 1 (as before); those with RdRp/MP NOL and MP/CP NOL are in Subgroup 2; those with both RdRp/MP OL and MP/CP OL are in Subgroup 3; and those with RdRp/MP NOL and MP/CP OL are in Subgroup 4 (Figure 4). We then compared these divisions to their phylogeny, based on a sequence of the nucleic acids, to determine the extent of relationships of these parameters to each other and to the family of the original hosts in which these viruses were discovered. These analyses are shown in Figure 5, from which the following observations can be made: (i) Those viruses grouped previously (by host plant of first isolation) in the Solanaceae family, are largely in Subgroup 1, as noted when there were much fewer tobamovirus sequences available, although several new ones are in Subgroup 2, in small clusters; viz., PMMoV and TSAMV, as well as PaMMV plus ObMV and SMMoV plus YTMMV. (ii) Conversely, some other viruses in Subgroup 1 (PluMV, FrMV, and OV2) are somewhat similar to each other, but quite distanced from the Solanaceae cluster. Moreover, the first two were isolated from a plant in the Apocynaceae family, while the third was isolated from a plant in the Cactaceae family. (iii) The orphan (recombinant) ORSV (isolated from a member of the family Orchidaceae) is in between the Solanaceae cluster and clusters involving other families. (iv) The cluster of tobamoviruses isolated from plants in the Brassicaceae family (TVCV, WMoV, and YoMV), along with the sole virus first isolated from a member of the Plantaginaceae family, RMV, are all in Subgroup 4. (v) By contrast, tobamoviruses isolated first from members of the Cactaceae family, although grouped by sequence identity, are spread over several Subgroups; viz., OV2 is in Subgroup 1, CMMoV is in Subgroup 3, whereas RCNaV, CTV1 and CTV2 are in Subgroup 4. (vi) Tobamoviruses that were found first in plants of the family Apocynaceae are grouped into two, small, adjacent clusters: HoNSV in Subgroup 4, together with HoCSV in Subgroup 3, and PluMV together with FrMV, both in Subgroup 1. (vii) The two tobamoviruses isolated first from *Plumeria rubra* (*f. acutifolia*) in the family Passifloraceae (MarMV and PFMV) are grouped together by homology and Subgroup—4. (viii) The two tobamoviruses isolated first from hibiscus plants in the family Malvaceae (HLFPV and HLSV) are grouped together by sequence and Subgroup—4. (ix) The two tobamoviruses isolated first from plants in the family Fabaceae (ClYMV and SHMV) are also grouped together by sequence homology and are both in Subgroup 3. (x) The next largest grouping after those tobamoviruses isolated first from plants in the family Solanaceae, are the seven viruses isolated first from plants in the family Cucurbitaceae, four of which (CFMMV, KGMMV, TrMMoV, and ZGMMV) form a continuum and are all in Subgroup 2, while the remaining three (WGMMV, CMoV, and CGMMV) extend the continuum but all are in Subgroup 3. (xi) The remaining tobamoviruses were first identified from single examples from novel plant families, viz., SFBV (Gesneriaceae) and UMMoV (Basellaceae) and are both in Subgroup 3. (xii) Some larger clusters of viruses that also show discovery from the same plant family, contain members found in two different Subgroups (e.g., from the families Solanaceae and Cucurbitaceae), whereas smaller clusters may contain either member from only one Subgroup (but more than one family of discovery, e.g., Brassicaceae/Plantaginaceae), or three different Subgroups (e.g., Cactaceae and Apocynaceae) (Figure 5). Thus, the value of subgrouping by whether particular ORFs overlap (or do not overlap) with each other may require further analysis to determine underlying patterns related to the adaptation of these viruses to particular hosts (e.g., [46]).

**Figure 6:**
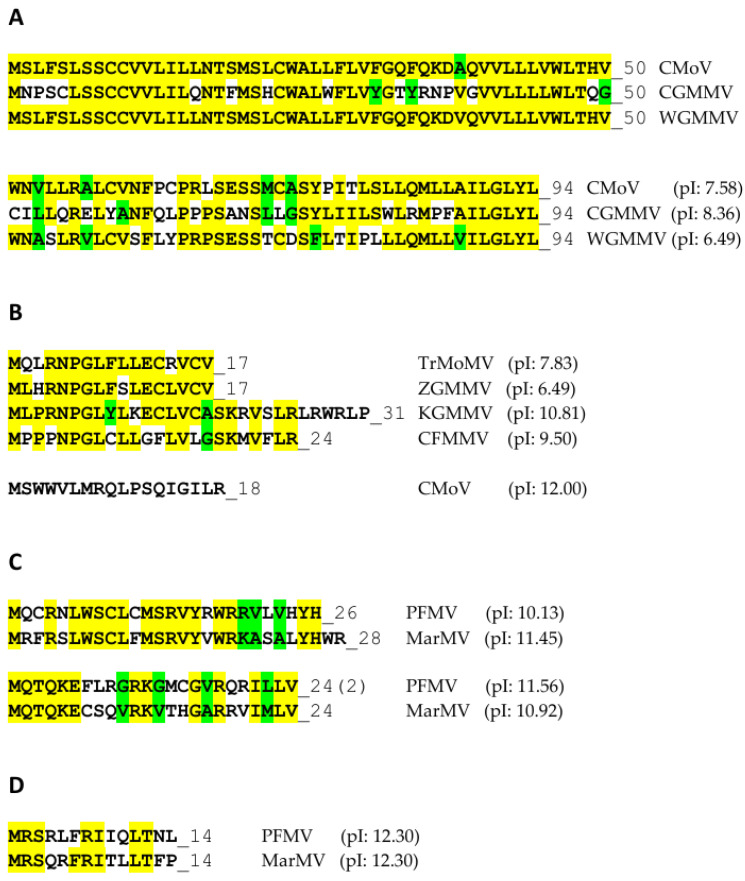
Conservation of sequences between putative P6 proteins derived from specific non-canonical ORFs 6 of different viruses in the same Subgroup and original host family cluster: (**A**) The sequence comparisons of Type B, 94-amino acid proteins encoded by three Subgroup 3 Cucurbitaceae-infecting tobamoviruses (CMoV, CGMMV, and WGMMV). (**B**) The sequence comparisons of Type B, shorter P6 proteins from similar locations in their respective genomes encoded by four Subgroup 2 Cucurbitaceae-infecting tobamoviruses (TrMoMV, ZGMMV, KGMMV, and CFMMV), vs. a similar-sized P6 protein encoded by a different region of CMoV. (**C**) The sequence comparisons of two sets of Type B, shorter P6 proteins from similar locations in their respective genomes encoded by two Subgroup 4 Passifloraceae-infecting tobamoviruses (PFMV and MarMV). (**D**) The sequence comparisons of Type D, shorter P6 proteins from similar locations in their respective genomes encoded by PFMV and MarMV. The pIs of the peptide or protein sequences also are shown. Amino acid sequences identical between compared proteins are shown in yellow highlight; those in which the amino acid sequences are similar, are highlighted in light green. Dissimilar amino acids are not highlighted.

The relationship of various putative P6 proteins to the virus phylogeny is not straightforward. Figure 3 shows that there was little conservation of size or sequence among those putative P6 proteins derived from Type A canonical ORFs 6, in Subgroups 1 and 2 (Table 2). By contrast, among the cucurbitaceous tobamoviruses, those viruses in Subgroup 3 (CMoV, CGMMV, and WGMMV), containing Type B ORFs 6 encoding putative proteins of 94 amino acids, showed high sequence identity to each other (Figure 6A), and were unrelated in sequence to the putative 75-amino acid protein encoded by Type D ORF6 of the Subgroup 2 ZGMMV, which was located further down in the genome (Table 2). Similarly, the small ORFs 6 of the four Subgroup 2 cucurbitaceous tobamoviruses (TrMoMV, ZGMMV, KGMMV, and CFMMV), encoding putative proteins of 17, 17, 31, and 24 amino acids, respectively, also were conserved highly in sequence, in their overlapping areas, but were unrelated in sequence to the small 18-amino acid putative protein of CMoV (Figure 6B), for which the Type B ORF6 also was located further downstream in the genome (Table 2). In numerous cases, small ORFs present in some related viruses were not present in others, since mutations in the virus genome within either the MP ORF or CP ORF, whether or not they also affected those coding sequences, could result in the loss of an initiation codon or a termination codon, the generation of an earlier termination codon, or the generation of a new in-frame upstream start codon, altering either the nature or the existence of a particular ORF. Hence, the two malvaceous tobamoviruses (HLFPV and HLSV), both in Subgroup 3, did not have any common ORFs (Table 2). This applied also to the two fabaceous tobamoviruses (ClYMV and SHMV) in Subgroup 3. By contrast, the two passifloraceous tobamoviruses (PFMV and MarMV), both in Subgroup 4, shared three ORFs in common (Table 2)—two Type B ORFs (Figure 6C) and one Type D ORF (Figure 6D)—in which there were considerable sequence similarities. Note also that many of these smaller putative P6 proteins have high pIs (Figure 6).

Although we have largely ignored ORFs 6 that encode putative peptides containing ≤15 amino acids, this was undertaken as a matter of convenience to reduce the number of putative P6 proteins under consideration. At some point, it may be worth exploring some of these shorter peptides for functions, given the number of very small plant peptides with biological activity [101,102,103,108]. In addition, various natural antimicrobial peptides (AMPs) are also described in the literature [107,108,130,131]. These are divided into various classes, based on their structures ([130] see legend to Figure 2). That work may also suggest a subject of further investigation for putative P6 proteins; viz., their three-dimensional structures.

## Figures and Tables

**Table 1:** Size range of P6 proteins > 15 amino acids (aa) among virus members of the genus Tobamovirus.

Subgroup	Virus Name (Abbrev.) & Accession No.	54 kDa	Species	P6 ORF
1	Bell pepper mottle virus (BPMV) DQ355023.1	+	+	*a* 25/16/18/36 aa
1	Brugmansia latent virus (BrLV) MK012556.1	+	−	*b*
1	Brugmansia mild mottle virus (BrMMV) AM398436.1	+	+	*a* 44/29/24 aa
3	Cactus mild mottle virus (CMMoV) EU043335	+	+	*a* 48/19 aa
4	Cactus tobamovirus 1 (CTV1) MW938767.1	+	−	*a* 18 aa; *c* 20 aa
4	Cactus tobamovirus 2 (CTV2) MW938769.1	+	−	*a* 29/30 aa; *c* 19 aa
1	Chili pepper mild mottle virus (ChPMMoV) MN164455.1	+	−	*a* 17/20/16/46 aa *d* 27 aa
3	Clitoria yellow mottle virus (ClYMV) JN566124	+	+	*a* 16 aa
2	Cucumber fruit mottle mosaic virus (CFMMV) AF321057.1	+	+	*a* 47/24/67 aa
3	Cucumber green mottle mosaic virus (CGMMV) D12505.1	+	+	*a* 94 aa
3	Cucumber mottle virus (CMoV) AB261167.1	+	+	*a* 94/18 aa
1	Frangipani mosaic virus (FrMV) HM026454.1	+	+	*b*
3	Hibiscus latent Fort Pierce virus (HLFPV) AB917427.1	+	+	*a* 67/16/26 aa *e* 22 aa
3	Hibiscus latent Singapore virus (HLSV) AF395898.3	+	+	*a* 20 aa
3	Hoya chlorotic spot virus (HoCSV) KX434725.1	+	+	*a* 26/22/31 aa *e* 21 aa
4	Hoya necrotic spot virus (HoNSV) MN961200.1	+	−	*a* 34/88 aa; *e* 20 aa
2	Kyuri green mottle mosaic virus (KGMMV) AJ295948.1	+	+	*a* 31 aa; *e* 21 aa
4	Maracuja mosaic virus (MarMV) DQ356949.1	+	+	*a* 18/28/24 aa *e* 14 aa
2	Obuda pepper virus (ObPV) D13438.1	+	+	*a* 19 aa
1	Odontoglossum ringspot virus (ORSV) X82130	+	+	*a* 22/42 aa; *d* 38 aa
1	Opuntia virus 2 (OV2) MF434821.2	+	−	*a* 31/18 aa; *e* 38 aa
2	Paprika mild mottle virus (PaMMV) AB089381	+	+	*a* 20 a.a.; *d* 51 a.a.
4	Passion fruit mosaic virus (PFMV) HQ389540.1	+	+	*a* 24/26/33/24 aa *e* 14 aa
2	Pepper mild mottle virus (PMMoV) M81413.1	+	+	*a* 21/27 aa
1	Plumeria mosaic virus (PluMV) KJ395757.1	+	+	*a* 24 aa
4	Rattail cactus necrosis-associated virus (RCNaV) JF729471.1	+	+	*a* 19/32 aa
1	Rehmannia mosaic virus (ReMV) EF375551.1	+	+	*a* 26/25 aa; *d* 51 aa
4	Ribgrass mosaic virus (RMV) HQ667979.1	+	+	*a* 19 aa; *f* (64 aa)
2	Scopolia mild mottle virus (SMMoV) LC643028	+	−	*a* 35 aa
3	Streptocarpus flower break virus (SFBV) AM040955.1	+	+	*a* 26 aa; *e* 55 aa
3	Sunn-hemp mosaic virus (SHMV) D84000.1	+	+	*a* 27 aa
*g* 2 or 4	Tobacco latent virus (TLV) AY137775	*g*	+	*a* 22/18 aa; *d* 36 aa
1	Tobacco mild green mosaic virus TMGMV) M34077.1	+	+	*d* 46 aa
1	Tobacco mosaic virus (TMV) V01408.1	+	+	*a* 51 aa; *d* 40 aa
1	Tomato brown rugose fruit virus (TBRFV) KT383474.1	+	+	*a* 22 aa; *d* 43 aa
1	Tomato mosaic virus (ToMV) AF332868.1	+	+	*a* 19/42 aa; *d* 33 aa
1	Tomato mottle mosaic virus (TMoMV) KF477193.1	+	+	*a* 26/39/62 aa *d* 43 aa
2	Trichosanthes mottle mosaic virus (TrMoMV) OL404963.1	+	−	*a* 20/17 aa; *e* 12 aa
2	Tropical soda apple mosaic virus (TSAMV) KU659022.1	+	+	*a* 21/57/42 aa *d* 35 aa
4	Turnip vein-clearing virus (TVCV) U03387.1	+	+	*a* 39 aa
3	Ullucus mild mottle virus (UMMoV) MH645158.1	+	+	*a* 16 aa
4	Wasabi mottle virus (WMoV) AB017503.1	+	+	*a* 31 aa; *h* (35 aa)
3	Watermelon green mottle mosaic virus (WGMMV) MH837097.1	+	−	*a* 94 aa
1	Yellow pepper mild mottle virus (YPMMoV) MN164454	+	−	*a* 17/28 aa; *d* 21 aa
2	Yellow tailflower mild mottle virus (YTMMoV) KF495564.1	+	+	*a* 19/24 aa
4	Youcai mosaic virus (YoMV) U30944.1	+	+	*a* 39 aa *i* (*a* 16 aa) *j* (*e* 63 aa
2	Zucchini green mottle mosaic virus (ZGMMV) AJ295949.1	+	+	*a* 17 aa; *e* 75 aa

Footnotes: *a* = Type B non-canonical ORF6 >15 aa (amino acids), completely inside MP ORF. *b* = Several short non-canonical ORFs ≤ 15 aa, inside MP ORF. *c* = Type C non-canonical ORF6, inside MP ORF that extensively overlaps CP ORF. *d* = Type A canonical ORF6, overlapping both non-overlapping MP and CP ORFs. *e* = Type D non-canonical ORF6, inside MP ORF, in-frame with and fused to CP ORF. *f* = Additional Type B non-canonical ORF6 found in one of three RMV strains. *g* = Unknown, due to only a partial sequence of MP ORF, CP ORF, and part of 3′ NTR. *h* = Two WMoV strains had a Type B ORF6 of 31 aa, while a third strain had 35 aa (different terminator). *i* = Type B non-canonical ORF6 found in most YoMV strains, but only 15 aa in ORMV strain U30944.1. *j* = Extension of 24 aa in Rg-strain of YoMV to the common 39 aa ORF in other YoMV strains, caused by termination codon mutation from UAA to UUA, leading to extension and fusion with CP ORF.

**Table 2:** Comparison of ORFs 6 location, size, subgrouping, and type for each tobamovirus.

SG *^a^*	Virus *^b^*	Location (nt) and Size (aa) *^c^*			
		Type A *^d^*	Type B *^e^*	Type C *^f^*	Type D *^g^*
1	TMV	5666–5788 (40)	5486–5641 (51)		
1	ReMV	5633–5788 (51)	5207–5287 (26); 5339–5416 (25)		
1	TBRFV	5671–5802 (43)	5455–5523 (22)		
1	TMoMV	5676–5807 (43)	4998–5078 (26); 5211–5330 (39); 5454–5642 (62)		
1	ToMV	5660–5761 (33)	4940–4999 (19); 5453–5581 (42)		
1	ChPMMoV	5654–5737 (27)	4916–4969 (17); 5189–5251 (20); 5276–5326 (16); 5486–5626 (46)		
1	BPMV		4884–4961 (25); 5110–5160 (16); 5268–5324 (18); 5478–5588 (36)		
1	YPMMoV	5648–5713 (21)	5279–5332 (17); 5486–5572 (28)		
2	PMMoV		5207–5272 (21); 5492–5575 (27)		
2	TSAMV	5634–5741 (35)	4929–4994 (21); 5208–5381 (57); 5448–5576 (42)		
1	BrMMV		4926–5060 (44); 5397–5486 (29); 5562–5636 (24)		
2	PaMMV	5693–5848 (51)	5199–5261 (20)		
2	ObPV		4952–5011 (19)		
2	SMMoV		5528–5635 (35)		
1	YTMMoV		4958–5017 (19); 5627–5701 (24)		
1	TMGMV	5626–5766 (46)			
1	ORSV	5634–5750 (38)	5076–5144 (22); 5463–5591 (42)		
4	HoNSV		5069–5173 (34);		5681–5740 (20)
3	HoCSV		5273–5353 (26); 5429–5497 (22); 5510–5602 (31)		5651–5713 (21)
3	SFBV		5395–5475 (26)		5506–5670 (55)
3	UMMoV		5001–5051 (16)		
4	TVCV		5417–5536 (39)		
4	RMV		4907–4966 (19)		
4	WMoV		5312–5419 (35)		
4	YoMV		5314–5364 (16); 5406–5525 (39)		
1	PluMV		5256–5330 (24)		
3	WGMMV		5212–5496 (94)		
3	CMoV		5214–5498 (94); 5613–5669 (18)		
3	CGMMV		5142–5426 (94)		
2	CFMMV		5149–5292 (47); 5464–5538 (24); 5629–5832 (67)		
2	KGMMV		5473–5568 (31)		5803–5865 (21)
2	TrMoMV		5396–5458 (20); 5486–5539 (17)		5840–5875 (12)
2	ZGMMV		5475–5528 (17)		5640–5864 (75)
1	OV2		5154–5249 (31); 5454–5510 (18)		5514–5628 (38)
4	RCNaV		5299–5358 (19); 5671–5769 (32)		
4	CTV2		5428–5514 (29); 5566–5655 (30)	5909–5968 (19)	
4	CTV1		5219–5275 (18)	5763–5825 (20)	
3	CMMoV		5255–5401 (48); 5408–5467 (19)		
3	HLFPV		5184–5387 (67); 5218–5268 (16); 5421–5501 (26)		5730–5795 (22)
3	HLSV		5545–5607 (20)		
3	ClYMV		5523–5573 (16)		
3	SHMV		5162–5245 (27)		
4	PFMV		5498–5572 (24); 5693–5773 (26); 5861–5962 (33); 5963–6037 (24)		6080–6121 (14)
4	MarMV		5541–5597 (18); 5697–5783 (28); 5967–6041 (24)		6084–6125 (14)

Footnotes: *a* = Subgroup number. *b* = Tobamovirus abbreviated name. *c* = Nucleotide (nt) positions of the ORFs in viral genomes and amino acid (aa) length of putative P6 proteins. *d* = Type A canonical ORF6, overlapping both non-overlapping MP and CP ORFs. *e* = Type B non-canonical ORF6 > 15 aa (amino acids), completely inside MP ORF. *f* = Type C non-canonical ORF6, inside MP ORF that extensively overlaps CP ORF. *g* = Type D non-canonical ORF6, inside MP ORF, in-frame with and fused to CP ORF.

## Data Availability

The data presented in this study are available in the article, as well as in online databases.
